# Evolution and a Creator

**Published:** 1991-09

**Authors:** M. G. Wilson


**EVOLUTION AND A CREATOR**
**Graham Beale**
Clinical Press £9.95

This book may be an important contribution to an argument developing in the scientific world about the origin of man. Darwin's theory of Evolution by Natural Selection was in its time a concept so heretical that it took Darwin, a Christian believer, many years before he could commit himself to expound it. It is now so universally accepted that to question it or to seek to modify it is almost equally heretical. Richard Dawkins, in his popular book *The Blind Watchmaker* has shown by computer studies that the operation of chance through aeons of time is sufficient to explain the development of man from the elementary matter of this universe. No need for a Creator. However of recent years a view has been gaining ground amongst some scientists notably in Britain and the USA which they have called the *Anthropic principle*. "There exist invariant properties of the Natural World and its elementary components which render the gross size and structure of virtually all its components quite inevitable. The sizes of stars and planets, even people are neither random nor the result of any Darwinian selection process from a myriad of possibilities.... they are manifestations of the possible equilibrium states between competing forces of attraction and repulsion.... and they are determined by a mysterious collection of pure numbers that we call the *Constants of Nature*. (Barrow and Tipler — The Anthropic Cosmological Principle, OUP 1986). The inference can be made that the Universe was designed, in fact that it was created.

From a study of the evolution of bone and the remarkable permanence of its basic design features Graham Beale argues that the gene determining it must have been present in the first cell and survived unchanged. Either this gene appeared by chance or it did not, if the latter, some other agency — *a genetic engineer* — must have formed it. Genetic uniqueness and the great genetic stability present in all aspects of all life cannot be explained in other ways.

Thus the Anthropic Principle postulates that the physical universe was designed and now Dr. Beale deduces from anatomical studies that the genetic system which governs heredity and thus man was also designed. Both conclusions seem to point to a Creator or "Architect of the Universe."

Dr. Beale's book is well illustrated with X-rays and his case well argued but his conclusion tentative, he ends by asking the question "If that is so, has evolution actually been the result of the action of a Creator? The book is easily readable and with 90 pages modestly priced.

Dr. Beale was a Radiologist who graduated in Medicine from the University of Otago, New Zealand and was Head of the Department of Radiology in Rotorua. Attracted orginally by the great reputation of the Bristol University Department of Radiology and its chief, Professor Sir Howard Middlemiss, Graham Beale has visited Bristol and worked there as a locum consultant periodically for a number of years.

The occasion of his last visit was the launching of his book, when your reviewer had the opportunity of meeting him and hearing him expound his ideas. His sudden death shortly after his return to New Zealand was a great shock to all who knew him and the loss of a profound and original thinker.

M. G. Wilson

